# Ultra-Narrow SPP Generation from Ag Grating †

**DOI:** 10.3390/s21216993

**Published:** 2021-10-21

**Authors:** Gerald Stocker, Jasmin Spettel, Thang Duy Dao, Andreas Tortschanoff, Reyhaneh Jannesari, Gerald Pühringer, Parviz Saeidi, Florian Dubois, Clement Fleury, Cristina Consani, Thomas Grille, Elmar Aschauer, Bernhard Jakoby

**Affiliations:** 1 Infineon Technologies Austria AG, 9500 Villach, Austria; Jasmin.Spettel@silicon-austria.com (J.S.); Thomas.Grille@infineon.com (T.G.); Elmar.Aschauer@infineon.com (E.A.); 2 Silicon Austria Labs GmbH, 9524 Villach, Austria; Thang.Dao@silicon-austria.com (T.D.D.); Andreas.Tortschanoff@silicon-austria.com (A.T.); Florian.Dubois@silicon-austria.com (F.D.); Clement.Fleury@silicon-austria.com (C.F.); Cristina.Consani@silicon-austria.com (C.C.); 3 Institute of Microelectronics and Micro Sensoric, Johannes Kepler University Linz, 4040 Linz, Austria; Reyhaneh.Jannesari@jku.at (R.J.); Gerald.Puehringer@jku.at (G.P.); Parviz.Saeidi@jku.at (P.S.); Bernhard.Jakoby@jku.at (B.J.)

**Keywords:** surface plasmon polaritons, refractive index sensing, reflection measurement, plasmonic grating

## Abstract

In this study, we investigate the potential of one-dimensional plasmonic grating structures to serve as a platform for, e.g., sensitive refractive index sensing. This is achieved by comparing numerical simulations to experimental results with respect to the excitation of surface plasmon polaritons (SPPs) in the mid-infrared region. The samples, silver-coated poly-silicon gratings, cover different grating depths in the range of 50 nm–375 nm. This variation of the depth, at a fixed grating geometry, allows the active tuning of the bandwidth of the SPP resonance according to the requirements of particular applications. The experimental setup employs a tunable quantum cascade laser (QCL) and allows the retrieval of angle-resolved experimental wavelength spectra to characterize the wavelength and angle dependence of the SPP resonance of the specular reflectance. The experimental results are in good agreement with the simulations. As a tendency, shallower gratings reveal narrower SPP resonances in reflection. In particular, we report on 2.9 nm full width at half maximum (FWHM) at a wavelength of 4.12 µm and a signal attenuation of 21%. According to a numerical investigation with respect to a change of the refractive index of the dielectric above the grating structure, a spectral shift of $4122\;\frac{\mathrm{nm}}{\mathrm{RIU}}$ can be expected, which translates to a figure of merit (FOM) of about 1421 ${\mathrm{RIU}}^{-1}$. The fabrication of the suggested structures is performed on eight-inch silicon substrates, entirely accomplished within an industrial fabrication environment using standard microfabrication processes. This in turn represents a decisive step towards plasmonic sensor technologies suitable for semiconductor mass-production.

## 1. Introduction

The number and variety of sensor applications has increased dramatically with the development of smart home systems. The sensing of the environment, for example ambient gases, is of growing interest for many applications such as indoor air quality monitoring, gas leakage or fire detection. The miniaturization of existing sensor concepts is one way to meet the demand of integrated sensors. In this context, plasmonics in the mid-infrared (MIR) region and their usage for sensor applications have emerged as a field of interest in the recent years [1]. These surface effects allow monitoring different physical or chemical properties at material interfaces in a non-invasive way [2,3,4,5] or even several physical properties at once [6,7]. Besides such sensing applications, the list of use-cases in the field of plasmonic research in the mid- and near-infrared region also covers waveguides [8], selective thermal emitters [9,10] and infrared (IR) detectors [11]. For all mentioned applications, the performance directly depends on the physical properties of the used materials and the structural geometry. These properties, together with the fabrication capabilities determine the usability in particular applications.

In this work, we numerically investigate and experimentally demonstrate the excitation of surface plasmon polaritons (SPPs) in the mid-infrared (MIR) region in silver-coated poly-silicon gratings. The study covers a wavelength range of 4.1 µm–4.3 µm which is of interest for gas-sensing applications, particularly for ${\mathrm{CO}}_{2}$ gas sensors. To demonstrate ultra-narrow SPP resonances, we probe plasmonic gratings of four different depths (50 nm, 150 nm, 225 nm, 375 nm), keeping the grating geometry fixed. A narrowband resonant plasmonic grating with high absorptivity would constitute an ideal platform for e.g., sensitive refractive index sensing [6,12]. Plasmonic gratings with a highly confined near-field at the metal-dielectric interface could be applied for surface-enhanced infrared absorption spectroscopy (SEIRA) [13]. In the presented work, the experimental characterization of the samples covers angle-resolved reflection measurements, where employing a tunable quantum cascade laser (QCL) adds another degree of freedom to the experiment. The findings indicate that by increasing the grating depths, the resonant bandwidth (FWHM) tends to increase while the resonant specular reflectance decreases (absorptivity increases). Therefore, the grating with 50 nm depth revealed the narrowest resonances, for example at 4.12 µm wavelength under a $28^{\circ }$ incidence with an FWHM of 1.4 nm by simulation and 2.9 nm by experiment. To show the potential of the suggested structure with respect to sensing applications, a numerical investigation concerning sensitive refractive index sensing is presented.

Besides the theoretical and experimental part, we also describe the fabrication of the samples done on eight-inch silicon wafers, entirely performed in a state-of-the-art clean room facility of semiconductor industry. In particular, the feasibility to fabricate plasmonic grating structures for the mid-infrared region within an industrial fabrication environment using standard microfabrication processes represents a decisive step towards plasmonic sensor technologies suitable for semiconductor mass-production.

Preliminary results of this work were presented at the Transducers 2021 Conference and published in its Proceedings [14].This extended paper is intended to provide further and deeper insights to the study.

The remainder of this work is organized as follows. Section 2 describes a short background of the SPP excitation using the grating configuration. This section also discusses the choice of silver as the preferential material for SPPs in the MIR region, as well as the optical measurement setup and the sample fabrication. The angle-measurement results are presented and discussed in Section 3 where we show the strategy how to control the grating depth for the narrowband SPP resonance. Additionally, sensitive refractive index sensing is numerically demonstrated as potential sensing application. Finally, Section 4 summarizes the work.

## 2. Materials and Methods

### 2.1. Surface Plasmon Polaritons

**Figure 1:**
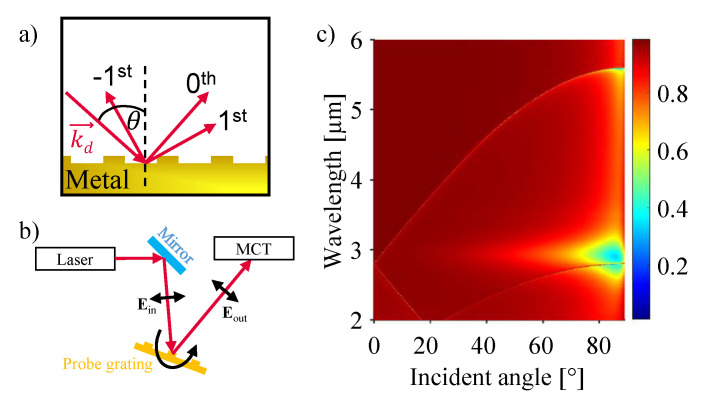
Reflection of radiation at a metal-dielectric interface. (**a**) Schematic of the reflection of a plane wave at a metal grating (dark yellow). The incident beam is represented by its wave vector $\vec{k_{d}}$ and incident angle θ. The 0th, 1st and −1st order of reflection are shown. (**b**) Schematic of the experimental setup. The laser light is guided via mirrors towards the sample grating. The angle of incidence can be adjusted by rotating the sample. The reflected light is collected by a mercury cadmium telluride (MCT) detector. The polarization is chosen perpendicular to the etched grating (TM polarized) and within the plane of incidence (p-polarized). (**c**) Surface plot depicting the simulated reflectivity of a silver grating as a function of wavelength and incident angle (0th order).

The presented and discussed measurements employ free-beam reflection measurements to probe plasmonic properties of our structures, which are designed to allow the excitation of SPPs at the silver-air interface. SPPs are the collective oscillations of free electrons within a metal-like layer introduced by the interaction with photons at the layer’s surface [15]. The resulting wave, propagating along the interface of dielectric (air) and plasmonic layer (silver), is of both photonic and plasmonic nature. The strength of the electric field is exponentially decaying from the interface into both media [16]. To excite a SPP by a photon, both need to have the same momentum. At a given frequency, a free space photon has less momentum than a SPP, due to different dispersion relations [15]. The wave number of the SPP, $k_{\mathrm{SPP}}$, is given by

(1) $$k_{\mathrm{SPP}}=k_{d}\sqrt{\frac{\epsilon_{d}\epsilon_{m}}{\epsilon_{d}+\epsilon_{m}}},$$

where $k_{d}$ is the wave number in the dielectric and $\epsilon_{d}$ and $\epsilon_{m}$ is the relative permittivity of the dielectric and the metal, respectively [15]. To overcome this momentum mismatch, coupling structures, for example prisms [17,18], or gratings [19] are needed, where in this work grating coupling is performed. For grating coupling the SPP’s momentum can be written as

(2) $$\hbar k_{\mathrm{SPP}}=\hbar k_{d}\sin\left(\theta \right)+\hbar n\frac{2\pi }{p},$$

where the surface-parallel component of the photon wave vector, $k_{d}\sin\left(\theta \right)$, is increased by an amount linked to the period *p* of the grating and the order *n* of diffraction [20]. Figure 1a depicts a schematic of the reflection of a laser beam at a metal grating. The actual reflection is composed of several orders corresponding to the different values of *n* with the 0th, 1st and −1st order indicated in the figure. It is important to note that Equation (2) states nothing about the intensity that is given to each mode. This intensity is dependent on the width and the depth of the corresponding grating structure [21]. Those parameters determine the loss rate by radiation of the SPP resonance and must be aligned to accomplish critical coupling [21,22,23]. Notably, critical coupling can be achieved with various choices of the grating width. Our choice for the duty cycle of 0.43 requires a grating depth of 150 nm for critical coupling. This in turn results in a convenient range of values for the grating depths (with respect to the fabrication), to study changes of the reflectance as a function of the grating height. Lower or higher duty cycles would require shallower or deeper gratings, respectively, to observe similar behavior of the reflectance and still observe critical coupling. To probe the samples an experimental setup as described in Figure 1b is employed (details given in Section 2.3). The surface plot in Figure 1c depicts the simulation results for a silicon grating (width = 1.6 µm, period = 2.8 µm, depth = 50 nm) coated with 100 nm silver. The area of reduced intensity of the reflected light indicates, for which combinations of wavelength and incident angle Equation (2) is fulfilled as the reduced intensity is caused by the excitation of SPPs at the metal surface (0th order). The corresponding literature values for the relative permittivity, for all simulations, are taken from [24].

### 2.2. Optical Properties

**Figure 2:**
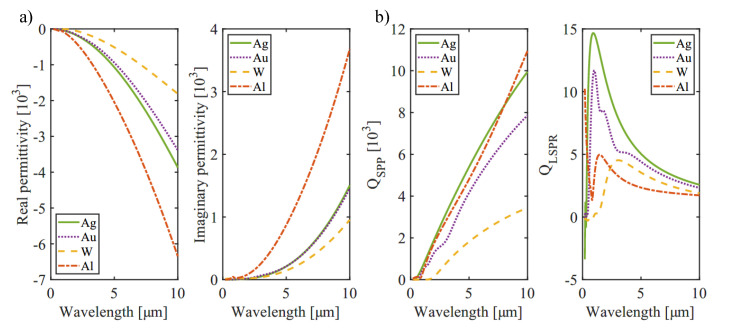
Optical properties of silver (Ag), gold (Au), tungsten (W) and aluminum (Al). (**a**) Real and imaginary part of the relative permittivity $\epsilon_{m}$. (**b**) Localized surface plasmon resonance quality factor $Q_{\mathrm{LSPR}}=\frac{-Re(\epsilon_{m})}{Im(\epsilon_{m})}$ and surface plasmon polariton quality factor $Q_{\mathrm{SPP}}=\frac{Re{\left(\epsilon_{m}\right)}^{2}}{Im(\epsilon_{m})}$ plotted for a wavelength range of 0–10 µm. Literature values are taken from [24].

In this work we investigate silver as plasmonic material. Figure 2 compares the optical properties of silver (Ag), gold (Au), tungsten (W) and aluminum (Al), namely the real and imaginary part of the relative permittivity $\epsilon_{m}$. The corresponding literature values for the near- and mid-infrared region are taken from [24]. A high negative real part of the permittivity is an indicator that plasmonic resonances should be observable. The comparison of the mentioned metals shows that silver and aluminum provide the most promising plasmonic properties. On the other hand, the imaginary part of the permittivity states how lossy the materials are. This value increases for increasing wavelength and is similar for gold and silver, with the lowest losses to be expected for tungsten. In this respect aluminum shows the highest losses among the compared materials.

For even better quantification, these optical properties can be used to calculate two typical quality-factors (Q-factors): One for surface plasmon polaritons ($Q_{\mathrm{SPP}}=\frac{Re{\left(\epsilon_{m}\right)}^{2}}{Im(\epsilon_{m})}$) and one for localized surface plasmon resonance ($Q_{\mathrm{LSPR}}=\frac{-Re(\epsilon_{m})}{Im(\epsilon_{m})}$), both illustrated in Figure 2b. The corresponding equations are valid for interfaces of metal and air [25].

Although a high value of $Q_{\mathrm{LSPR}}$ correlates to strong plasmon resonance observable, $Q_{\mathrm{SPP}}$ can be used to calculate the propagation length for SPPs at a certain wavelength. The propagation length $L_{\mathrm{SPP}}$ is given by

(3) $$L_{\mathrm{SPP}}=\frac{c}{\omega }{\left(\frac{Re\left(\epsilon_{m}\right)+\epsilon_{d}}{Re\left(\epsilon_{m}\right)\cdot \epsilon_{d}}\right)}^{\frac{3}{2}}\frac{Re{\left(\epsilon_{m}\right)}^{2}}{Im(\epsilon_{m})},$$

where *c* is the speed of light in vacuum and ω is the angular frequency of the photon [16]. In the regime of $\epsilon_{d}=1$ and $|\epsilon_{m}|\gg \epsilon_{d}$, the equation can be simplified to

(4) $$L_{\mathrm{SPP}}\approx \frac{c}{\omega }Q_{\mathrm{SPP}}.$$

As depicted in Figure 2b, silver has the highest values for both Q-factors and is therefore considered to be a promising candidate to observe SPPs. For a wavelength of 4.26 µm, which is of interest in the context of ${\mathrm{CO}}_{2}$ sensing, the calculated propagation length is around 990 µm. Depending on the actual use-case this constitutes a limitation for possible applications.

### 2.3. Experimental Setup and Sample Fabrication

The experimental setup used to characterize the samples is built according to the schematic given in Figure 1b. The mid-infrared light source is a tunable quantum cascade laser (QCL, MIRcat${}^{\mathrm{TM}}$, DRS Daylight Solutions, San Diego, CA, USA), built in a setup for free-beam reflection measurements. Wavelength sweeps are performed in pulsed mode from 3.96 µm to 4.35 µm, covering the whole spectrum of the QCL. The samples are mounted on a manual rotation stage, to control the angle of incidence. The beam position and polarization are controlled by a periscope placed between laser and sample. The polarization is chosen perpendicular to the etched grating (TM polarized) and within the plane of incidence (p-polarized). The 0th order of reflection is guided to a mercury cadmium telluride (MCT, PVI-4TE-6, VIGO System, Poznan, Poland ) detector. A lock-in amplifier (SR830 DSP, Stanford Research Systems, Sunnyvale, CA, USA) and an oscilloscope (PicoScope, 5000 Series, Pico Technology, Cambridgeshire, UK) are used to perform the detector read out.

**Figure 3:**
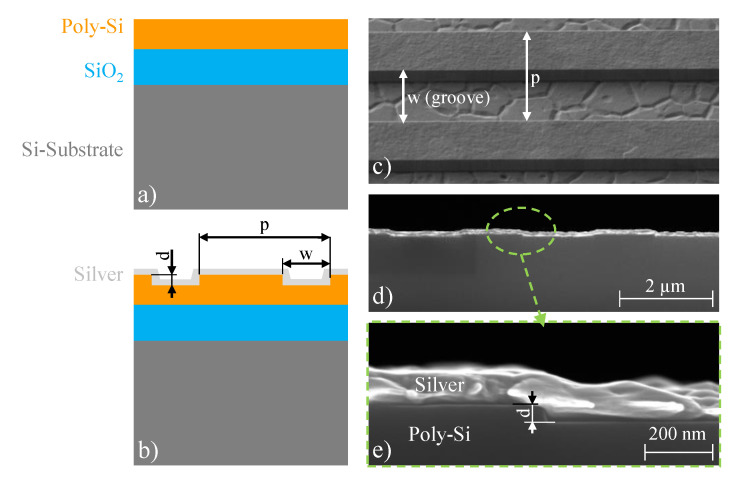
Samples for the layer characterization. (**a**) Schematic of the layer stack used for fabrication. The eight-inch silicon substrate (dark gray) is covered by around 2 µm of silicon oxide (${\mathrm{SiO}}_{2}$, blue) and 600 nm of poly-silicon (Poly-Si, orange). (**b**) The poly-Si is structured by means of lithography and dry etching. The poly-Si grating is covered with silver (light gray). The dimensions drawn are the depth *d*, the width *w* and the period *p* of the grating. (**c**) Scanning electron microscope (SEM) image of etched grating structures (50 nm depth, *p* = 2.8 µm, *w* = 1.6 µm). The SEM is taken before metallization with a tilt of $45^{\circ }$. (**d**) SEM cross-section picture of a similar grating structure as shown in (**c**), uniformly covered by 100 nm silver (evaporated). (**e**) Close-up of the region indicated by the green-dashed ellipse in (**d**).

The samples are fabricated on eight-inch silicon-substrate wafers in the clean room facilities of Infineon Technologies Austria AG in Villach. A schematic of the layer stack is given in Figure 3a. The silicon substrate is covered by around 2 µm of silicon oxide (${\mathrm{SiO}}_{2}$) for decoupling of the grating structures from the substrate. On top of the ${\mathrm{SiO}}_{2}$, 600 nm of poly-silicon (poly-Si) is deposited. The actual grating is introduced to the poly-Si by means of lithography and a corresponding dry-etch process. The process duration is adjusted to fabricate gratings of 50 nm, 150 nm, 225 nm and 375 nm depth. After etching and resist removal, the gratings are covered by silver as shown in the schematic of Figure 3b. The scanning electron microscope (SEM) image in Figure 3c depicts the etched grating (50 nm depth) before metallization. The large grains visible in the grooves is the typical appearance of plasma-etched poly-Si. To have a uniform metal coating, also at the sidewalls of the gratings, the 50 nm grating is metallized by means of evaporation, whereas for all deeper grating structures sputtering is performed. A SEM image of the cross-section of grating structures with 50 nm depth, covered with 100 nm silver (evaporated) is given in Figure 3d, with a close-up in Figure 3e.

## 3. Results and Discussion

**Figure 4:**
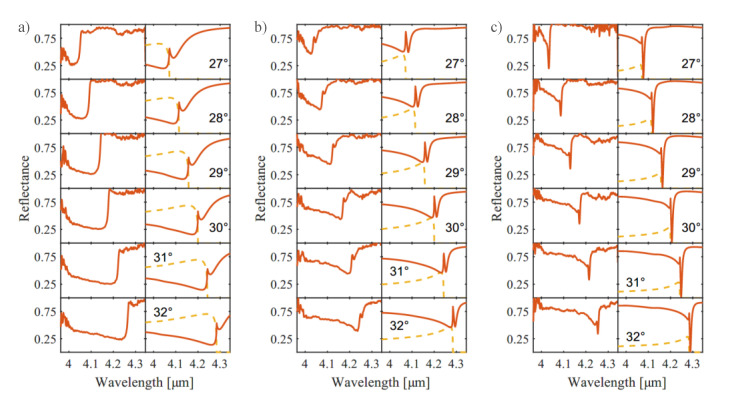
Measurement results and simulations for different grating depths. Each left panel represents the measured intensity of the 0th order of reflection (red). The simulated data are given in the right panels, consisting of the corresponding 0th (red) and the −1st (orange, dashed) order. The tested samples are silver-coated (100 nm, sputtered) grating structures of (**a**) 375 nm, (**b**) 225 nm and (**c**) 150 nm depth. The incident angle of the laser beam with respect to the sample surface is varied from $27^{\circ }$ to $32^{\circ }$. The wavelength is tuned from 3.96 µm to 4.35 µm.

With the experimental setup described above, we investigated the wavelength and angular dependence of the reflection on the different samples. The wavelength of the QCL can be adjusted with such an accuracy that no error contribution from wavelength uncertainty is included in the discussion. The incident angle is varied by manually rotating the sample and the error is estimated to be $\pm 1^{\circ }$. The retrieved data are compared to simulations, carried out with the rigorous coupled-wave analysis (RCWA) (DiffractMOD package, Synopsys’ RSoft, Mountain View, CA, USA). For all measurements presented in this section the QCL covers the same wavelength range from 3.96 µm to 4.35 µm. In Figures 4, 6 and 7, the panels on the left represent measured values, whereas the right one depicts results retrieved from simulation. For wavelengths beyond 4.25 µm, the absorption of ${\mathrm{CO}}_{2}$ present in the ambient air is perceptible in the data. The presented data are connected to the preliminary first experimental results published in [26].

Figure 4 depicts the results of grating structures of 375 nm, 225 nm and 150 nm depth. The incident angle is varied from $27^{\circ }$ to $32^{\circ }$. According to Equation (2), the resonance wavelength for SPP coupling increases as the incident angle does. This can be seen in the data as the resonance dip shifts by around 220 nm for an angle change of $5^{\circ }$. The sharp dip in the reflectance spectra corresponds to the SPP resonance, whereas the broader feature at shorter wavelength, results from diffraction into the −1st order. Especially for grating depths 375 nm and 225 nm (Figure 4a,b), the intensity of the −1st order (orange, dashed line in the right panels) of reflection cannot be neglected for wavelengths shorter than the one of SPP resonance. For the grating with 150 nm depth (Figure 4c) the intensity of the −1st order of reflection becomes much weaker. As a result, the decrease in intensity due to the SPP resonance becomes much more striking. At $27^{\circ }$ of incident angle, the measured intensity of the reflected light has a level of around 80% at a wavelength of 4 µm. The intensity drops to close 65% at 4.020 µm wavelength. The center of the feature is located at 4.036 µm with a full width at half maximum (FWHM) of 5.5 nm and a minimum intensity of around 20%. For wavelengths beyond 4.046 µm the intensity approaches 100%. The FWHM and center position of the resonance are retrieved from fitting the data with a Fano model [27,28]. The same analysis can be performed for the simulated data. For $27^{\circ }$ incidence the simulated data predict the center at 4.077 µm with a FWHM of 5.2 nm and a vanishing intensity.

The weaker attenuation observable in the measured data with respect to the simulation has several reasons: besides roughness of the silver layers and potential worse optical properties of the deposited Ag films, not perfectly polarized laser light can have an influence. All simulations assume perfect transverse magnetic (TM) polarization of the incident light with infinite grating periods. For deeper grating structures, the resonances become wider, which agrees with the theoretical expectation [21].

**Figure 5:**
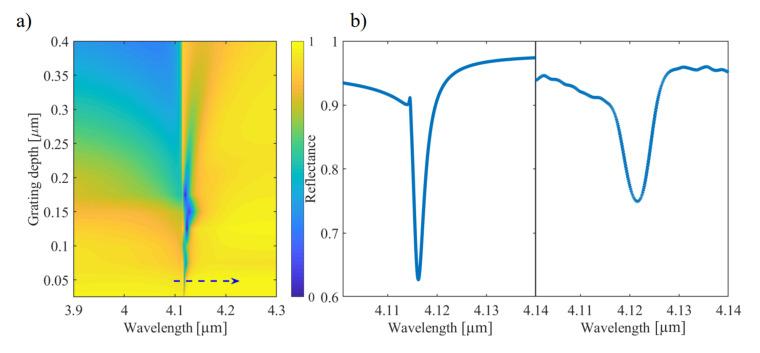
Optimization of grating depth to retrieve ultra-narrow SPP resonances for potential sensing applications. (**a**) Simulated dependence of 0th order reflectance (specular reflectance) at $28^{\circ }$ on the grating depth. (**b**) Simulated (left panel) and measured (right panel) specular reflectance at $28^{\circ }$ of the grating of 50 nm depth (*p* = 2.8 µm, *w* = 1.6 µm).

As a tendency, reducing the grating depth results in narrower SPP resonances. To use this effect to shape the line width of the resonance we perform additional numerical investigation. Figure 5a depicts the simulated dependence for the 0th order of reflection (specular reflection) on the grating depth. For the target wavelength range of 3.96 µm to 4.35 µm, a grating depth of 50 nm is predicted to reveal resonances in the range of 1.4 nm FWHM and a signal attenuation of around 30%, for an angle of incidence of $28^{\circ }$ (see Figure 5b left panel). Figure 5b represents a comparison of the simulated (left panel) and measured (right panel) SPP resonance feature in reflection for the grating of 50 nm depth at an incident angle of $28^{\circ }$.

**Figure 6:**
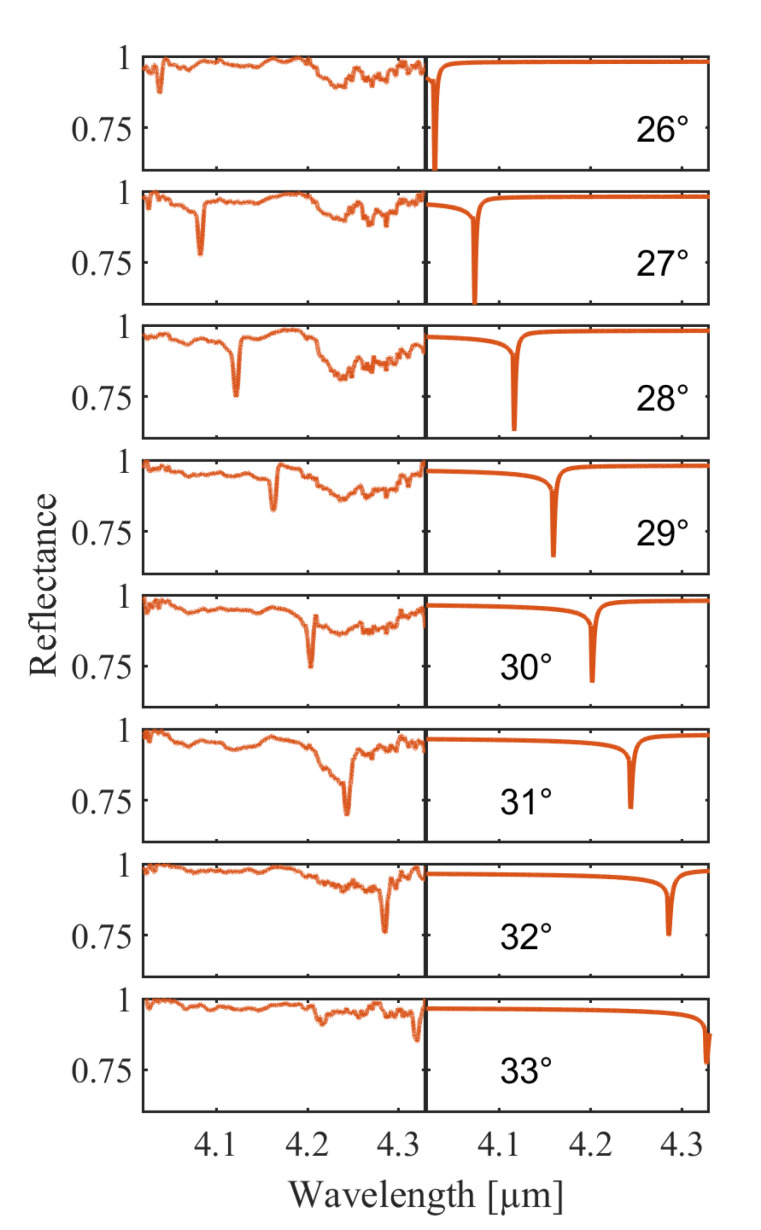
Comparison of measurement results (**left panel**) and simulated data (**right panel**) for a silver-coated (100 nm, evaporated) grating of 50 nm depth. The incident angle of the laser beam with respect to the sample surface is varied from $26^{\circ }$ to $33^{\circ }$ and the wavelength is tuned from 3.96 µm to 4.35 µm. The panels represent a reflectance of 60–100%.

Therefore, we further investigate a shallower silver grating of 50 nm depth. In Figure 6, a similar comparison of measured values and simulation is depicted. The measurement covers an angle variation in the range of $26^{\circ }$ to $33^{\circ }$. The resonance dip shifts by around 280 nm for an angle change of $7^{\circ }$. The SPP resonance at $28^{\circ }$ of incident angle reduces the intensity of the reflected light from close 96% (off-resonant) to a level of around 75% which translates to an attenuation of 21%. The feature has a FWHM of 2.9 nm and is centered at a wavelength of 4.122 µm. The simulation reveals a FWHM of 1.4 nm with the resonance center at 4.116 µm and a minimum intensity of 63%.

**Figure 7:**
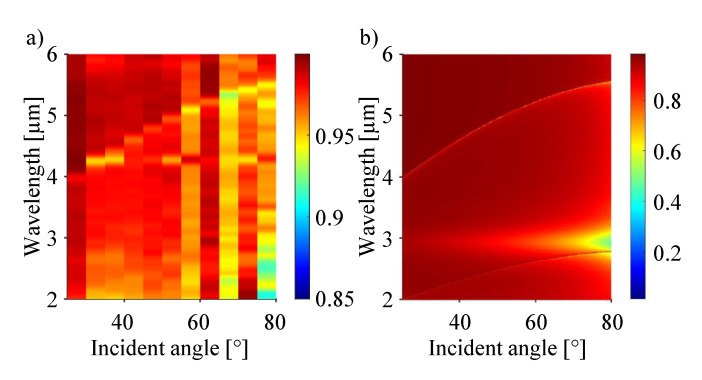
Surface plot depicting the reflectivity of a grating with 50 nm depth, coated with 100 nm silver (evaporated). Plotted is the wavelength and incident angle for the 0th order of reflection. The areas of reduced intensity are due to the excitation of SPPs at the metal’s surface. (**a**,**b**) Representation of measured values and a corresponding simulation, respectively.

The surface plots in Figure 7 illustrate the specular reflectance of the same grating sample of 50 nm depth coated with silver in a wider range of incident angles and wavelengths. The wavelength regime for this measurement reaches from 2 µm to 6 µm and the angle of incidence is varied from $25^{\circ }$ to $80^{\circ }$ with a step-size of $5^{\circ }$. The measured and simulated values are given in Figure 7a,b, respectively. As already mentioned and explained for Figure 1c, the reduced reflection is caused by the excitation of SPPs at the interface. The lines of reduced intensity within these plots indicate, for which combinations of wavelength and incident angle Equation (2) is fulfilled. The measurement is done with a FTIR ellipsometer (IR-VASE, J.A. Woollam Co., Lincoln, NE, USA). Even if the absolute values of reflected intensity are different, the measured values (left panel) are in good agreement with the simulated ones (right panel). Since the excitation beam in the FTIR ellipsometer is not a coherent light source, the signal to noise ratio is lower (shallower resonance with higher noise floor) compared to the reflectance measurement using the QCL. The main source of noise within the experimental setup is the unwanted absorption of the light by ${\mathrm{CO}}_{2}$ and water vapor present in the laboratory’s atmosphere. The bright, almost angle independent feature at 3 µm is attributed to the higher order of the localized surface plasmon mode.

### Potential Applications for Sensing

**Figure 8:**
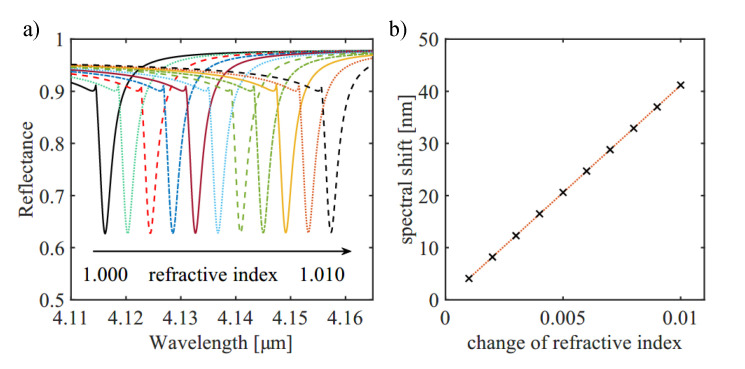
Numerical investigation of refractive index sensing based on the shallow grating structures of 50 nm depths. (**a**) Simulation of the spectral shift of the SPP resonance due to slight changes of the refractive index *n* of the dielectric. The refractive index is changed from 1.000 refractive index unit (RIU) to 1.010 RIU. (**b**) From the simulation in (**a**), an average spectral shift of $4122\;\frac{\mathrm{nm}}{\mathrm{RIU}}$ can be determined.

The presented measurements and the material properties of plasmonic layers in general allow various sensing applications based on plasmonics and SPPs. Previous works suggest different use-cases, for example thin-film measurements or refractive index sensing [4,21,29]. As an example, we numerically investigate the possibility to use these structures as a platform for refractive index sensing. Changes in the refractive index in the cladding introduce spectral shifts in the SPP resonance. For high sensitivity, ultra-narrow SPP resonances are needed, which can be retrieved by optimizing the grating parameters. As suggested by the simulation presented in the beginning, we indeed see such ultra-narrow SPP resonances for the grating with 50 nm depth at an incident angle of $28^{\circ }$ (see Figure 5a,b). Further numerical investigations concerning refractive index sensing with respect to our grating samples (50 nm depth) are depicted in Figure 8a,b. Changes of the refractive index of the dielectric between 1 and 1.010 refractive index units (RIUs) can be resolved with an average spectral shift of $4122\;\frac{\mathrm{nm}}{\mathrm{RIU}}$. In the field of refractive index sensing, tuning the plasmon line shape is used to narrow down the line widths. This in turn results in a higher figure of merit ($FOM=\frac{\frac{d\lambda }{dn}}{FWHM}$) and therefore lowers the detection limit [30]. In our case the FOM is around 1421 ${\mathrm{RIU}}^{-1}$ [12,30,31]. However, comparing the retrieved results for the grating with 150 nm and 50 nm depth, it can be seen that there is an optimum depth at a given period, for which the amplitude of the resonance reaches its maximum. Even though the grating with 50 nm depth reveals narrower resonances, the grating with 150 nm depth would enable a higher signal to noise ratio achievable for a sensor application. The mentioned range from 1 to 1.010 RIU was used only to determine the differential sensitivity of the proposed structure and it does not correspond to a meaningful change in the ambient atmosphere.

## 4. Conclusions

In summary, we have successfully demonstrated the excitation of ultra-narrow SPP resonances from plasmonic, metalized, thin-film gratings in the MIR region for, e.g., sensing applications. Motivated by simulations on optical properties of different metals, we identified silver as a proper material of choice, since the optical properties of this metal are promising with respect to the excitation of SPPs in the MIR region. The fabricated samples covered different grating depths, 50 nm, 150 nm, 225 nm and 375 nm, which allows active shaping of the line width of the SPP resonance. We shortly discussed the theoretical background and corresponding numerical calculations. The experimental part covered the wavelength range from 3.96 µm to 4.35 µm in the mid-infrared part of the spectrum. The retrieved experimental data are in good agreement with the simulations carried out. This is found for the layer properties in general, the dependence of the resonance on the angle of incidence as well as on the wavelength. The expected SPP resonances can be observed as a reduced intensity of the specular reflectance. In particular, the grating with a depth of 50 nm revealed SPP resonances with a FWHM of 2.9 nm and a signal attenuation of 21%. SPP resonances of this kind, with an ultra-narrow line width and a pronounced signal attenuation, are needed to be of use for practical sensing applications. In this context, we further investigated the feasibility to use the suggested grating structures as a platform for refractive index sensing. Indeed, the calculated sensitivity of a sensor based on the grating with a depth of 50 nm is found as high as $4122\;\frac{\mathrm{nm}}{\mathrm{RIU}}$ (FOM=1421 ${\mathrm{RIU}}^{-1}$). In addition to the promising results from the experiments and simulations presented, which show that the examined samples represent an interesting platform for future sensor applications, we would like to state that the entire fabrication took place in the industrial clean room facilities of Infineon Technologies Austria AG. Therefore, this work also proofs that plasmonic sensor technologies are compatible with the standard processes and mass production of the state-of-the-art semiconductor industry.

## Data Availability

Not applicable.
